# Modulation of Two-Photon Absorption Due to the Reversible Change in the Oxidation State of Atomically Precise Gold Nanoclusters

**DOI:** 10.1021/acs.jpclett.5c03956

**Published:** 2026-03-12

**Authors:** Patryk Obstarczyk, Julia Osmolska, Martina Perić Bakulić, Antonija Mravak, Marek Samoc, Joanna Olesiak-Banska

**Affiliations:** † Institute of Advanced Materials, Faculty of Chemistry, Wroclaw University of Science and Technology, Wybrzeze Wyspianskiego 27, 50-370 Wroclaw, Poland; ‡ Faculty of Chemistry and Technology, 74422 University of Split, Ruđera Boškovića 35, 21 000 Split, Croatia

## Abstract

The atomically precise nanoclusters offer precise control of optical properties on the nanoscale, as long as we understand the relation among the size, composition, and structure of nanoclusters and their physical properties. So far, the primary interest has been in the synthesis of nanoclusters with controlled optical properties, in not only the one-photon but also the two-photon regime. Large two-photon absorption cross sections of nanoclusters were reported, but the possibility of modulation of nonlinear optical (NLO) properties by external stimuli has been scarcely explored. We present here the experimental data supported by DFT calculations on two-photon absorption of gold nanoclusters, which are modified by oxidation–reduction processes. Such reactions can be an important regulator of the nonlinear optical properties of various materials, which is well established, e.g., for organometallics, but has not been demonstrated for nanoclusters. The reversible oxidation of [Au_25_(SR)_18_]^−^ (where SR = 2-phenylethanethiol) to its neutral form results in distinct changes in the one-photon absorption spectra, but even more pronounced differences in the two-photon absorption. The change in the oxidation state of the Au_25_ cluster results in the 2-fold enhancement of the two-photon absorption cross sections in the wavelength range of 825–1150 nm and switching between saturable absorption and two-photon absorption below 825 nm. DFT calculations show that the presence of the counterion may contribute to the change, as it decreases the two-photon absorption cross sections of the system. Our results demonstrate that even seemingly minor electronic differences between the anionic and neutral Au_25_(PET)_18_ clusters can lead to pronounced variations in their NLO properties.

## Graphical abstract

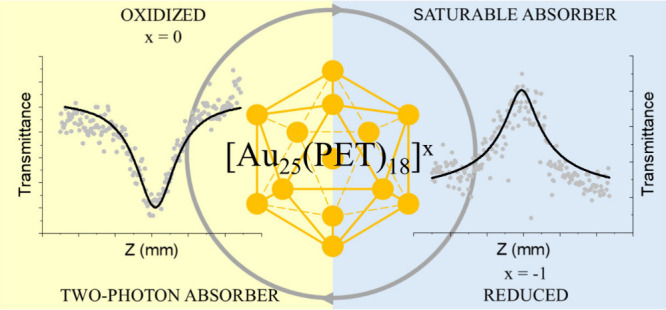

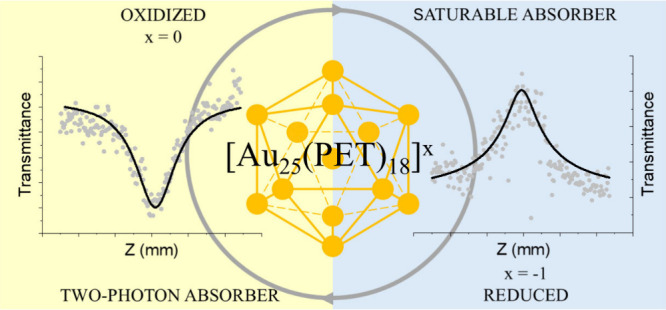

Gold nanoclusters (NCs) can be defined as nanoparticles with diameters of <2 nm that are too small to support localized surface plasmons but, instead, display quantum confinement effects manifested by the presence of distinct bands in their absorption spectra,^1^ and therefore, they constitute a bridge between molecular and bulk crystal structures.^2^ Lately, the structural and electronic properties of ligand-protected and atomically precise NCs, i.e., ones with a well-defined number of gold atoms and ligands, have received much attention. They present unique optical characteristics: near-infrared (NIR) luminescence, high photostability, large Stokes shifts, and long lifetimes of excited states.^3,4^ Moreover, gold nanoclusters were found to be robust probes for applications in two-photon imaging;^5,6^ their two-photon absorption cross sections (σ_2_) are typically orders of magnitude larger than those reported for commonly used organic dyes,^6–8^ and the nanoclusters present relatively high values of the two-photon absorption figures of merit (σ_2_/*M*). However, comprehensive studies of their nonlinear optical properties are still scarce in the literature, and systematic approaches are of great importance.^7,9–11^

NCs with a formula of Au_25_(SR)_18_, where R is an organic group, have been widely investigated because of their extraordinary stability, among other reasons.^12^ These NCs are composed of an icosahedral Au_13_ core that is protected by six staple-like motifs with a general chemical formula of Au_2_(SR)_3_.^4^ Numerous computational and experimental works have been conducted on Au_25_(SR)_18_ stabilized with the 2-phenylethanethiol ligand (S­(CH_2_)_2_Ph, PET).^13,14^ These clusters can be obtained in the neutral or anionic state, i.e., [Au_25_(PET)_18_] or [Au_25_(PET)_18_]^−^, respectively, which can also be termed the oxidized and reduced forms of the cluster. Their crystal structures were resolved, and structural distortion was detected for anionic NCs.^15^ One of the sulfur atoms in the Au_2_(SR)_3_ motif is bent downward, while the inverted sulfur is bent upward due to the presence of the counterion, i.e., tetraethylammonium cation ([TOA]^+^). The oxidized clusters were found to have in-plane localization of the aforementioned sulfur atoms. However, the distortion in the nanocluster structure may be reconsidered at three different levels, NCs core, staple-like motifs, and ligands,^16,17^ which distinctively correspond to their optical properties. The anionic NCs possess a noble gas-like electron configuration (1s^2^1p^6^), and their oxidized counterparts are characterized by an unpaired 1p electron (1s^2^1p^5^).^16^ Nevertheless, the cluster framework is maintained in both oxidation states, as the main absorption bands at 400, 450, and 670 nm were detected for the anionic and neutral states.^12,15^ However, oxidation also introduces changes in the absorption spectrum: a decrease in the 450 band with a simultaneous increase in the 400 nm band and the disappearance of a transition at 800 nm.

To date, no studies have focused on differences regarding the nonlinear optical (NLO) properties of NCs in reference to their oxidation state. Switching the NLO properties by changing the oxidation state of a metal in mono- or bimetallic organometallic molecules and dendrimers has been well established,^18–20^ and drastic changes in the nonlinear absorption behavior have been observed, e.g., a transition from a two-photon absorber to a saturable absorber. However, since the differences identified in one-photon absorption of Au_25_ clusters at distinct oxidation states are not as profound as in the case of small molecules, the question is the degree to which the oxidation state also influences the two-photon absorption spectra of the NCs. Moreover, the two-photon properties of [Au_25_(SR)_18_]^−^ have been presented in the literature, but in the case of [Au_25_(SR)_18_] nanoclusters, they have not been reported (see Table S1).

In this work, we present a wide wavelength range (725–1150 nm) comparison of the two-photon properties of neutral Au_25_(SR)_18_ nanoclusters stabilized by the PET ligand with those of their anionic form. The NLO characteristics of [Au_25_(PET)_18_] and [Au_25_(PET)_18_]^−^ were determined for the clusters dissolved in toluene using the Z-scan technique and employing low-repetition rate femtosecond laser excitation. To complement the experimental results, density functional theory (DFT) geometry optimizations and time-dependent DFT (TDDFT) calculations were carried out to evaluate the nonlinear optical properties. We indicate that, indeed, the oxidation state of and presence of a counterion of Au_25_(SR)_18_ NCs have an important impact on their nonlinear optical properties; i.e., they strongly influence the two-photon absorption cross sections in the range of 825–1150 nm. Moreover, below 825 nm, the phenomenon of switching from a two-photon absorber to a saturable absorber was observed for the anionic clusters.

[Au_25_(PET)_18_]^−^ and [Au_25_(PET)_18_] NCs were successfully synthesized (see Methods), and their one-photon absorption profiles were found to present a series of characteristic and clearly resolved bands at 670, 450, and 400 nm (Figure 1). These experimental data are in good agreement with the literature, where the ground state one-photon extinction spectra of [Au_25_(PET)_18_]^−^ and [Au_25_(PET)_18_] are presented.^13,16^ The main absorption bands maintained in both forms confirm that the Au_25_ cluster framework is well preserved. Nevertheless, the anionic clusters do contain some additional spectral features, i.e., a broad shoulder band at 800 nm and another band at 550 nm. The aforementioned shoulder bands are not visible in [Au_25_(PET)_18_], where the peak at 400 nm is more prominent and a shoulder at 620 nm appears. The observed one-photon absorption spectra are consistent with the literature.^13,15,16^

**Figure 1:**
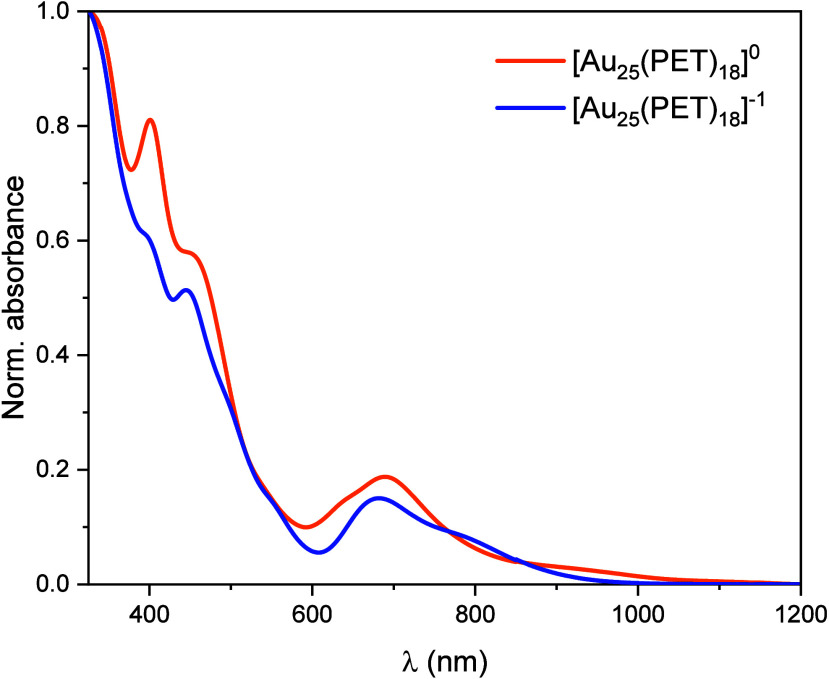
Normalized absorption of [Au_25_(PET)_18_] (orange) and [Au_25_(PET)_18_]^−^ (blue) in toluene.

Figure 2 presents the experimentally determined two-photon absorption spectra together with the corresponding one-photon absorption (1PA) of both samples and 1PA replotted at twice the wavelength. This way of presenting 2PA results is quite common in the case of two-photon absorbing dyes and has the advantage of allowing one to verify whether the 1PA and 2PA processes involve the same final excited state. However, the overlap of the 2PA spectrum with 1PA replotted against 2λ is not expected where symmetry-based selection rules make 1PA reachable states forbidden for 2PA (and the other way round) or when the 2PA process is not a direct instantaneous transition, but it involves sequential absorption with the participation of a real intermediate state. Also, when several excited states participate in the spectra, the relative magnitudes of the corresponding peaks seen in 1PA and 2PA do not have to be the same.

**Figure 2:**
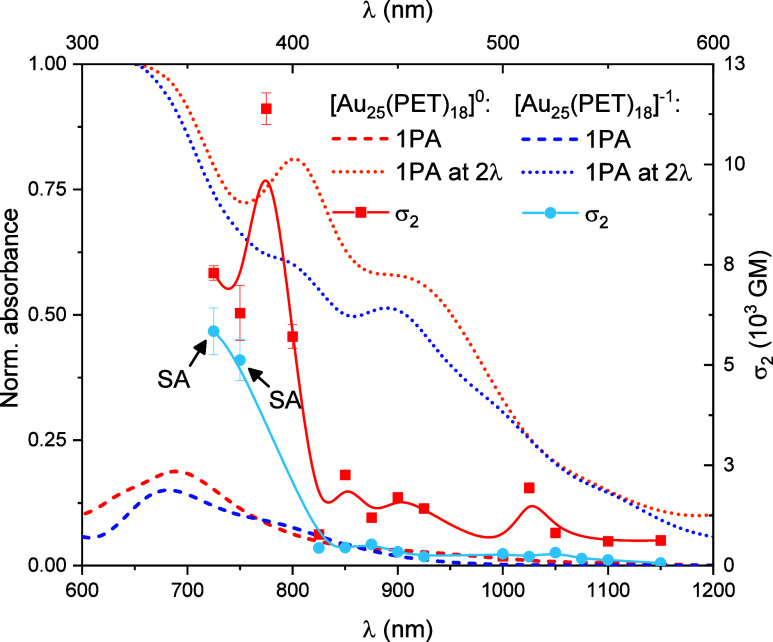
Calculated off- and in-resonance two-photon absorption cross sections (σ_2_) in Goeppert–Meyer (GM) units of Au_25_(PET)_18_ gold nanoclusters at the −1 (light blue filled circles) and 0 (red filled squares) oxidation states, connected by B-spline lines to guide the eye. Corresponding one-photon absorption (1PA, blue and red dashed lines, respectively) and one-photon absorption at twice the wavelength (1PA at 2λ, blue and orange dotted lines, respectively) are also presented. Data obtained for distinctive Z-scan traces identified as 2PA in the presence of saturated absorption (SA) are marked with black arrows. The upper *x*-axis scale was introduced to help assess whether the state reached by 2PA excitation can also be accessed via 1PA at the corresponding wavelengths.

Based on Figure 2, two distinct spectral regions can be distinguished for the sake of the comparison of σ_2_ values for oxidized and neutral Au_25_(PET)_18_, namely, 725–950 and 950–1150 nm. The aforementioned regions were distinguished due to the presence (below 950 nm) and absence (above 950 nm) of significant one-photon absorption. Significant σ_2_ values were observed in the range of 725–950 nm. These wavelengths overlap with a shoulder of the one-photon absorption band, which has a maximum at 670 nm. The highest σ_2_ value was determined for [Au_25_(PET)_18_] (11 400 ± 400 GM at 775 nm). The corresponding value for the anionic clusters could not be unambiguously determined, as below 825 nm the Z-scan curves registered for [Au_25_(PET)_18_]^−^ showed a distinctive increase in the vicinity of *z* = 0, which can be explained by combination of the saturation of the absorption process with a two-photon absorption contribution (see Figure S1). In this case, both σ_2_ and the absorption saturation intensity need to be given to define the observed nonlinear response. The *I*_sat_ values were 80 and 65 GW/cm^2^ with the corresponding σ_2_ values equal to 5840 and 5120 GM at 725 and 750 nm, respectively. Application of the same fitting procedure for [Au_25_(PET)_18_] allowed us to conclude that the *I*_sat_ values in that case are >300 GW/cm^2^ for both wavelengths. However, for the corresponding wavelengths, the [Au_25_(PET)_18_] sample is characterized by two-photon absorption with σ_2_ values equal to 7290 ± 180 and 6297 ± 680 GM, respectively. In the second region, i.e., 950–1150 nm, [Au_25_(PET)_18_] is generally characterized by up to 2 times larger two-photon absorption cross sections in comparison to those of [Au_25_(PET)_18_]^−^.

Au_25_SR_18_^–^ is an eight-electron shell closing compound.^21^ The extra electron in [Au_25_(PET)_18_]^−^ results in a closed shell configuration, while [Au_25_(PET)_18_] is an open shell system; therefore, the optical and magnetic behaviors of both are distinctive. Crystal structures of anionic and neutral forms of Au_25_(PET)_18_ were presented by Zhou et al.^15^ Au_25_(SR)_18_ clusters are composed of several shells of symmetrically related atoms that can be distorted up to a distinct degree with a change in the oxidation state (e.g., core, staple-like motifs, and ligands). A notably higher-resolution crystal structure of Au_25_(PET)_18_ was reported by Tofanelli et al.,^16^ in which distortions from idealized symmetry of the icosahedral NCs core decreased in the reduced form of Au_25_(PET)_18_, in comparison to the oxidized one. Overall, one can assign both forms (anionic and neutral) of Au_25_(PET)_18_ to be roughly centrosymmetric, with slightly more deviation from the ideal symmetry in the oxidized form. It may be noted that the 2PA peak positions presented in Figure 2 are not perfectly aligned with those for 1PA plotted at 2λ. This feature may be expected for centrosymmetric systems, although the selection rules should be cautiously translated into this system due to its complexity.

In order to resolve the origin of the observed differences between 2PA of anionic and neutral clusters, our experimental results were supported by computational chemistry calculations. It is important to note that the electronic structure of [Au_25_(PET)_18_]^−^ can be modified by the presence of charged counterion [TOA]^+^, which might alter the orientation of PET ligands and introduce some distortion into the Au_13_ core.^15^ Moreover, as shown by Tofanelli et al.,^16^ as measured by CMS (continuous symmetry measure), the presence of a counterion forces deviations from ideal cluster symmetry, as the coordinates of sulfur atoms in the outer shell octahedron have the larger average dihedral angle in the reduced state, in comparison to that of the oxidized cluster.

To gain deeper insight into the correlation between the presence of a counterion and two-photon absorption properties, the polarizable embedding quantum mechanics within the time-dependent density functional theory (PEQM-TDDFT) method was employed (see the Supporting Information for details). In this approach, Au_25_(SCH_3_)_18_ represents the quantum mechanical (QM) region of the ligand-protected nanocluster. The effects of the surrounding environment, including the 18 remaining PET ligands and the counterion, are incorporated through effective operators that define an embedding potential. This potential accounts for both electrostatic and polarization interactions between the QM system and its environment, thereby directly influencing the molecular properties of the core system. The wave function of the QM part is optimized self-consistently, explicitly considering electrostatic effects from the environment. The calculations were designed to capture the local perturbation imposed by a counterion that remains in close contact with the ligand shell, as expected in low-dielectricity solvents such as toluene. Accordingly, the AuNC–TOA^+^ gas-phase complex should be viewed as a limiting-case representation of a contact ion pair, not as a model for a fully solvated, solvent-separated ionic species.

As shown in Figure 3, inclusion of the [TOA]^+^ counterion leads to a pronounced quenching of the two-photon absorption (TPA) signal, decreasing the cross section from approximately 10^8^ to 8 × 10^6^ GM, in both cases the maximum 2PA being located at around 670–680 nm, which is in excellent agreement with our experimental results. This observation is somewhat unexpected, as counterion-induced rigidification of the cluster shell is typically associated with an enhancement of the two-photon absorption cross section. A possible explanation is that the interaction of [TOA]^+^ with the surface ligands alters the local electronic environment and screening around the metal core, reducing electronic delocalization rather than promoting structural rigidity. According to previous first-principles studies, the positive charge on Au atoms within the icosahedral core increases with the oxidation state of the cluster, leading to a weakened electron-withdrawing ability of the sulfur atoms.^17^ As a result, the S atoms may withdraw less electron density (from the core), which may decrease 2PA efficiency. Thus, qualitative as well as quantitative differences in σ_2_ values are expected.

**Figure 3:**
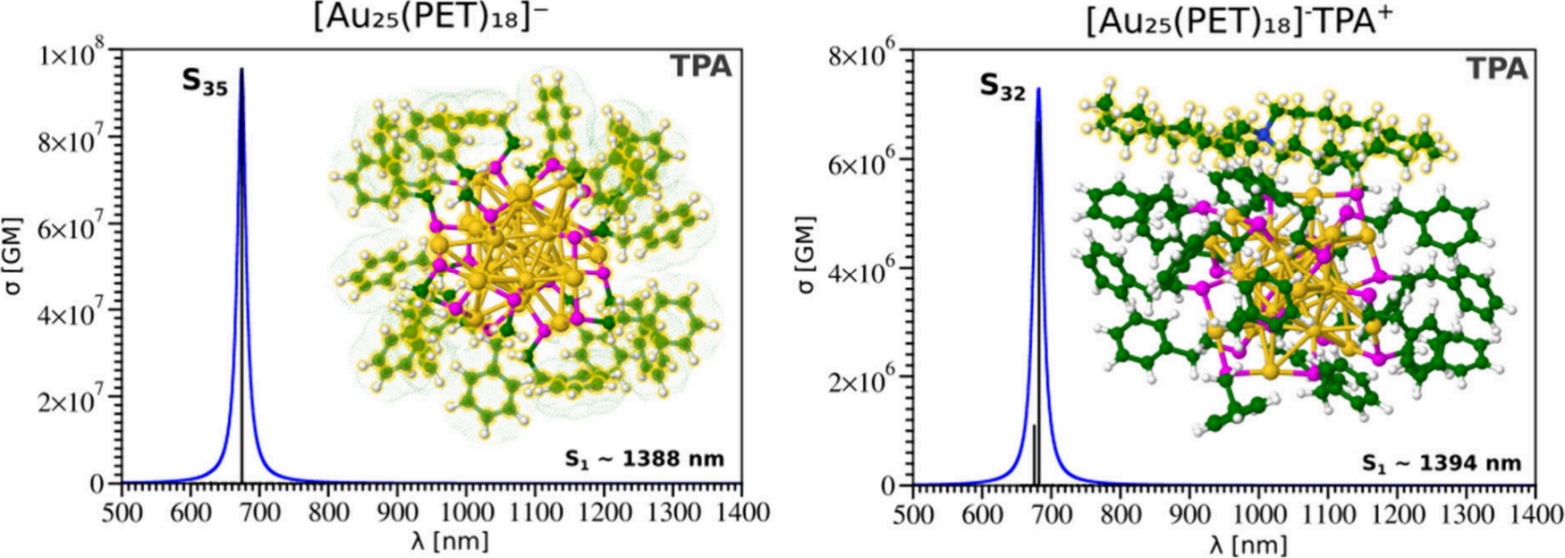
Comparison of the TDDFT-calculated two-photon absorption cross sections of [Au_25_(PET)_18_]^−^ clusters in the presence and absence of the [TOA]^+^ counterion. The top inset shows the DFT-optimized structure of [Au_25_(PET)_18_]^−^, where gold atoms are colored yellow, sulfur atoms of the PET ligands purple, carbon atoms green, and hydrogen atoms white.

High 2PA of the oxidized form at <800 nm may be associated with a resonance effect with the 1.6–1.8 eV (β′) transition, which, according to calculations,^17^ is absent for the anionic form. Several studies also indicate that the transition near 3 eV (HOMO–5/LUMO) conserves parity (g → g).^22^ Therefore, at the corresponding wavelength of ∼800 nm, the oxidized form is characterized by strong 2PA, while for the anionic form, a respective transition may be (i) not allowed or (ii) not enhanced due to the lack of the resonance effect with 1PA. To resolve the influence of the oxidation of the electronic spectrum of nanoclusters, three systems were modeled: [Au_25_(SCH_3_)_18_]^−^ (anion, singlet), [Au_25_(SCH_3_)_18_O_2_]^−^ (anion with one O_2_, triplet), and [Au_25_(SCH_3_)_18_] (neutral, doublet-unrestricted open shell) (see Figure S2). The 1PA of the neutral species calculated with TDDFT (green line in Figure S2) does not reproduce the experimental spectrum, nor does it resemble that of the calculated anionic species. However, the simulation performed in the presence of triplet O_2_ indicates that the neutral species likely coexist or interact with molecular oxygen under the experimental conditions. Consequently, the oxidation mechanism of the Au_25_(PET)_18_ clusters remains ambiguous.

To gain deeper insight into the role of cluster charge, their two-photon absorption (2PA) properties were investigated theoretically. To reduce the computational cost while preserving the essential electronic features, the PET ligands were replaced by methyl groups. The electronic influence of the O_2_ molecule was approximated using a point charge representation, in which a negative point charge was placed at the position corresponding to the O_2_ molecule in the optimized DFT geometry of the Au_25_(SCH_3_)_18_O_2_ model system (see Figure 4).

**Figure 4:**
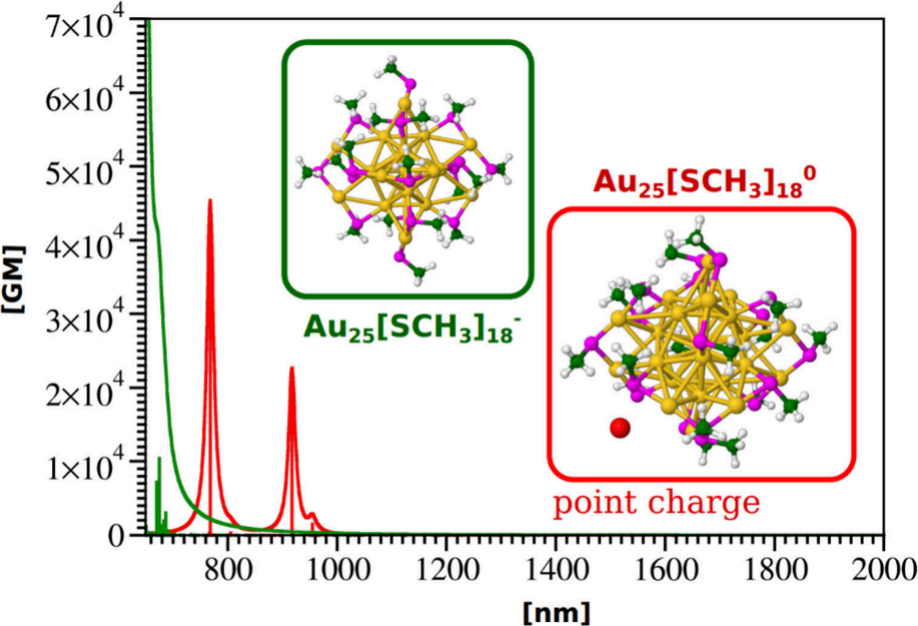
Comparison of the TDDFT-calculated two-photon absorption cross sections of Au_25_(SCH_3_)_18_ clusters depending on the charge state. The top inset shows the optimized structures of [Au_25_(SCH_3_)_18_]^−^ and [Au_25_(SCH_3_)_18_], where gold atoms are colored yellow, sulfur atoms in the SCH_3_ ligands purple, carbon atoms green, and hydrogen atoms white. The position of the point charge is indicated in red.

The calculated results are in good agreement with our experimental observations, showing that the neutral clusters exhibit larger two-photon absorption (TPA) cross sections in the range of 800–1100 nm compared to their anionic counterparts. However, one should note that the interaction of O_2_ with clusters remains ambiguous. We emphasize that this simplified treatment of O_2_ does not describe triplet O_2_ energy transfer, which has been shown to occur efficiently only for anionic Au_25_(SR)_18_^–^ clusters.^23^ Kauffma et al. identified two weakly bound states between O_2_ and the Au_25_(SCH_3_)_18_^–^ active site. According to their DFT-based studies, the O_2_ molecule was found to bind to three shell sulfur atoms or one shell gold atom, within the so-called adsorption pocket.^24^ Moreover, up to three oxygen adducts to anionic Au_25_(SR)_18_ were detected by mass spectrometry by Bhat et al.,^25^ which revealed that the first two O_2_ molecules interact with the surface gold atoms through the adsorption pockets formed by the ligands and staples while the third O_2_ interacts with only 2-phenylethanethiol ligands. The observed near-infrared (NIR) shift in the TDDFT-calculated spectra is most likely a consequence of the simplified computational model rather than intrinsic structural differences between the neutral and anionic clusters. In particular, the replacement of PET ligands with methyl groups significantly alters the electronic environment of the cluster. Substituting PET ligands with short SCH_3_ groups reduces the polarizability of the surrounding medium and the extent of electronic delocalization at the metal–ligand interface, which can result in shifts in the excitation bands. Additionally, representing the O_2_ molecule by a static point charge introduces an approximate electrostatic perturbation that does not fully capture charge-transfer or spin-polarization effects associated with explicit O_2_ coordination. This simplification can modify the potential energy landscape around the cluster and shift the calculated excitation energies.

Overall, the order of magnitude of the σ_2_ values presented in this work is in good agreement with measurements performed for Au_25_(Capt)_18_^–^, in water,^26^ as well as with theoretical calculations presented herein. The double resonance effect or excited state absorption may be the reason for the increased σ_2_ values^27,28^ in the range of 725–950 nm. Nevertheless, as presented in Figure 2, for both forms of NCs, the 1PA absorption is significant up to 950 nm. The anionic form presents absorption comparable to that of the oxidized form in the range of 750–850 nm; thus, it is not impossible that saturable absorption may be a process responsible for 2PA variation between oxidized and anionic NCs. The distinctive OA transmittance curves with characteristic peaks registered for the anionic form are consistent with the assumption that saturable absorption is dominant at the corresponding wavelengths (below 825 nm), contrary to the behavior of the oxidized clusters, where 2PA is clearly present. The order of magnitude of the *I*_sat_ values reported herein is in agreement with the data from our previous work, where the Au_25_(Capt)_18_^–^ system was studied.^26^

The presence of saturable absorption behavior in the spectral range below 825 nm is not unexpected because of substantial 1PA in that region. Indeed, spectral ranges where saturable absorption competes with two-photon absorption and with excited state absorption are often observed in various materials.^29^ It should be mentioned that a simple saturation model predicts that inverse saturation intensity *I*_sat_^–1^ should scale with the absorption coefficient, but the data at hand are too sparse to discuss the wavelength dependence of *I*_sat_ in the present case.

Our study of two-photon absorption of anionic and neutral Au_25_ nanoclusters stabilized with 2-phenylethanethiol carried out in the wavelength range of 725–1150 nm indicates that the oxidation state has a strong influence on the nonlinear optical properties of the clusters. This includes the enhancement of two-photon absorption cross sections (for the neutral form) and switching to a saturable absorber (for the anionic form). The NLO response of the NCs in the 0 and −1 oxidation states can differ by a factor of 5. The highest σ_2_ values, equal to 11 389 ± 392 GM (at 775 nm) and 11 680 ± 1168 GM (at 725 nm), were measured for the neutral and ionic clusters, respectively. Contrary to the oxidized form, the absorption spectra of anionic NCs are characterized by two 1PA shoulder bands. The distinct differences in σ_2_ values could be partly due to the presence of saturable or reverse saturable absorption, as these processes compete with direct two-photon absorption. We indicated that the anionic clusters, contrary to the two-photon absorbing oxidized counterparts, are characterized by saturable absorption in the range of 800–725 nm. Although the TDDFT calculations of the nonlinear optical (NLO) properties provide valuable insight into the electronic origin of the observed response, they do not fully reproduce the experimental differences between the oxidized and neutral clusters. The differences in NLO behavior may stem from subtle charge redistribution within the metal core and variations in the coupling between the core and the staple-like motifs, which can strongly influence the two-photon transition probabilities. Therefore, although the theoretical calculations successfully capture key electronic trends, the complete description of the NLO response requires consideration of both structural and environmental effects at multiple levels of the cluster framework. Above 800 nm, the σ_2_ values of the anionic clusters were also found to be several times lower, in comparison to those of the neutral form. We hypothesize that the double resonance effect and intertwined one-photon transitions may enhance differences in the NLO response of NC redox systems, within the entire wavelength range presented in this work.

Further computational chemistry investigations of the excited states of both cluster species would be valuable for clarifying the influence of geometrical relaxation and structural reorganization on the optical response in different oxidation states. Our results demonstrate that even seemingly minor electronic differences between the anionic and neutral Au_25_(PET)_18_ clusters can lead to pronounced variations in their NLO properties. Thus, it is possible to modulate the two-photon absorption of Au_25_(SR)_18_ NCs via reversible oxidation in the whole spectrum covered by the present studies, both in the range of the 1PA tail and at longer wavelengths (above 950 nm). Moreover, below 800 nm, the switching of the sign of the NLO response was revealed. This work therefore contributes to a better understanding of the NLO properties of atomically precise nanoclusters designed for functional applications in photonics.

## Supplementary Material



## Data Availability

The data sets generated and/or analyzed during the current study are available from the corresponding author upon reasonable request.

## Notes

Synthetic procedures for anionic and neutral Au_25_(PET)_18_ nanoclusters; oxidation methodology; UV–vis and fluorescence measurement details; nonlinear optical Z-scan setup and data analysis procedure; computational methods for DFT, TDDFT, and quadratic-response 2PA calculations; fitting procedure for open-aperture Z-scan curves; representative OA Z-scan traces; TDDFT-calculated 1PA spectra and structures; and tabulated experimental and simulated two-photon absorption cross sections (PDF)
