# Supplementary material for: Modulation of Two-Photon Absorption Due to the Reversible Change in the Oxidation State of Atomically Precise Gold Nanoclusters
Source: J Phys Chem Lett. 2026 Mar 12;17(12):3444–9. doi: 10.1021/acs.jpclett.5c03956 (PMC13298913; doi:10.1021/acs.jpclett.5c03956)
Supplement: Supplementary file 1 [file jz5c03956_si_001.pdf]

Modulation of two-photon absorption due to the reversible change of the oxidation state in atomically-precise gold nanoclusters

Patryk Obstarczyk[1], Julia Osmolska[1], Martina Perić Bakulić[2], Antonija Mravak[2], Marek Samoc[1], Joanna Olesiak-Banska[1*]

[1] – Institute of Advanced Materials, Faculty of Chemistry, Wroclaw University of Science and Technology, Wybrzeze Wyspianskiego 27, 50-370 Wroclaw, Poland.

2 - Faculty of Chemistry and Technology, University of Split, Ruđera Boškovića 35, 21 000 Split, Croatia.

* - Corresponding author joanna.olesiak@pwr.edu.pl

Methods

Materials

Gold (III) chloride trihydrate ($HAuCl_4$ x $3H_2O$, ≥ 99.9 %), 2-phenylethanethiol ($C_6H_5CH_2CH_2SH$, 98%), tetra-n-octyl ammonium bromide ($Oct_4N^+Br^-$, 98%) sodium borohydride ($NaBH_4$, ≥ 98.0 %), Styryl 9M ($C_{27}H_{31}ClN_2O_4S$, dye content ~ 96%), tetrahydrofuran (C_4H_8O, suitable for HPLC, ≥99.9%, inhibitor-free), toluene ($C_6H_5CH_3$, anhydrous, 99.8%) methanol (CH_3OH, suitable for HPLC, ≥ 99.9%), dichloromethane (CH_2Cl_2, suitable for HPLC, ≥ 99.8%, containing amylene as stabilizer) and silica gel (SiO_2, high-purity grade, w/ Ca, ~0.1 %, pore size 60 Å, 230-400 mesh particle size, for column chromatography) were used without further purification, as purchased from Sigma Aldrich. High-purity water (Milli-Q quality) was used throughout all the experiments.

$[Au_{25}(PET)_{18}]^-[TOA]^+$ synthesis and its oxidation

Gold nanoclusters at -I oxidation state were synthesized according to the protocol proposed by Parker *et al.*[1], with slight modifications. Methodology to oxidize clusters was based on Tofanelli *et al.*[2] and Zhu *et al.*[3] work. In short, gold (III) chloride trihydrate (1 g) and tetra-n-octyl ammonium bromide (1.56 g) were dissolved in tetrahydrofuran (70 mL) and stirred for 30 min (in a 200 mL round bottom flask). After that, 2-phenylethane thiol (1.8 mL) was pipetted into the mixture and stirred for additional 4 h until the resulting solution turned from faint yellow to colorless. In the meantime, ice-cold aqueous solution of sodium borohydride solution was prepared (0.965 g in 24 mL of Mili-Q water), sonicated (1 min) and rapidly added to the vigorously stirred (1200 rpm) THF mixture. The reaction flask was loosely capped (to prevent THF evaporation) and left for two days, gently stirred (600 rpm). After that, reaction product was gravity filtered to remove the insoluble materials. The THF solution containing gold nanoclusters was dried under vacuum and remaining oily residue was dissolved in 50 mL of toluene and transferred into a separatory funnel. In the next step, excess of the counter-ion was removed by extracting with water (100 mL). Aqueous phase was subsequently removed and remaining organic layer was dried. The resulting oily product was washed thoroughly with methanol, until characteristic smell of thiols was no longer present, leaving pure $[Au_{25}(PET)_{18}]^-[TOA]^+$. Part of $[Au_{25}(PET)_{18}]^-[TOA]^+$ was dissolved in the minimal amount of dichloromethane (DCM) and run through the column (solvent: DCM, stationary phase: SiO_2) to obtain clusters in its oxidized form. After processing, the collected fraction was dried and fine powder containing $Au_{25}(PET)_{18}$ at 0 oxidation state was obtained. For further studies, both clusters species were dissolved in toluene.

UV-Vis and fluorescence measurements

Absorption spectra were recorded with a Jasco V-670 spectrophotometer.

Nonlinear optical measurements

For the Z-scan experiment, the transmittance was measured as the cuvette with solution of anionic or neutral NCs (in toluene) was travelling along focused laser beam (*via* the focal point). In our experimental set-up, an open-aperture (OA) and closed-aperture (CA) traces were measured simultaneously and analyzed using the equations from Sheik-Bahae *et al.*[4] and procedures described in Samoć *et al.*[5] work. Exemplary OA transmittance curves, as well as corresponding fits are available in the supporting information (see figure S1).

The Z-scan transmittance curves (closed-aperture (CA), open-aperture (OA) and reference) were measured simultaneously using three InGaAs photodetectors (Thor Laboratories Inc.) for 900 – 1200 nm and three Si photodetectors (Thor Laboratories Inc.) for 750 – 900 nm wavelength range, respectively. A fs Ti:Sapphire amplifier Astrella (Coherent) laser combined with a OPA TOPAS Prime (Coherent) optical parametric amplifier were used as an excitation source to deliver tunable excitation wavelengths ranging from 750 nm to 1200 nm with pulse duration < 100 fs and repetition rate of 1 kHz. The output from the femtosecond pulsed laser was filtered with appropriate wavelengths color glass filters and attenuated with a neutral density filter to intensities ranging from 10 to 250 GW per cm^2. The excitation beam was focused to a focal point characterized by the beam waist ranging from 25 to 55 μm. Samples were placed in 1 mm path length Starna glass cuvettes and stoppered. A cuvette containing a pure solvent (toluene) and a silica plate used as a reference were subsequently measured under identical conditions. The experimental data was collected by a National Instruments PCI-6143 I/O card with the simultaneous sampling of all channels synchronized with the laser, and the data were transferred to a computer and analyzed using a custom-written LabVIEW software (supported by MatLab scripts), comprehensively described in our previous publications[6,7] and based on theory developed by Sheik-Bahae[4]. The fitting of open aperture traces in the case of coexistence of several nonlinear absorption mechanisms is described in the SI.

Computational details

Structural properties of $[Au_{25}PET_{18}]^-$, $Au_{25}PET_{18}$-TOA and $[Au_{25}(SCH_3)_{18}O_2]^-$ were investigated using density functional theory (DFT) with the PBE functional[8,9] as implemented in the Gaussian software package[10]. Initial coordinates were taken from the crystal structure[11] of $[TOA^+][Au_{25}(SCH_2CH_2Ph)_{18}^-]$. For initial coordinates of $Au_{25}(SCH_3)_{18}O^{2-}$ previously obtained DFT structure was used and adapted by replacing PET ligands with $-SCH_3$[12]. For gold atoms, the Stuttgart relativistic effective core potential (RECP) was employed[13]. The SVP basis set[14] was used for gold and sulfur atoms, while the 3-21G basis set[15,16], was applied to the remaining ligand atoms. For DFT/TDDFT calculations on models with shortened ligands only SVP basis set was used. Excited-state properties were computed within the TDDFT framework, using the Coulomb-attenuated version of Becke's three-parameter exchange functional combined with the Lee–Yang–Parr correlation functional (CAM-B3LYP)[17]. Singlet excited states were evaluated for $[Au_{25}PET_{18}]^-$, and $[Au_{25}PET_{18}]$TOA, while triplet states were considered for $[Au_{25}(SCH_3)_{18}O_2]^-$.
Two-photon absorption (2PA) properties were investigated using model with shortened ligands - methyl groups, resulting in the model system $[Au_{25}(SCH_3)_{18}]^-$. In order to account for the electronic effect of O_2 molecule the point-charge approximation was employed by placing a negative point charge at the position of the calculated geometry for O_2 from $[Au_{25}(SCH_3)_{18}O_2]^-$ model system. The O_2 environment is represented by a point charge to mimic an external electrostatic perturbation; this approximation does not account for explicit charge-transfer or spin-dependent interactions known to govern triplet-O_2 sensitization in anionic $Au_{25}(SR)_{18}^-$ clusters[18] .The 2PA calculations were carried out within the framework of quadratic response theory[19] as implemented in the Dalton quantum chemistry program. For the calculation of 2PA properties of $[Au_{25}(PET)_{18}]^-$ and $[Au_{25}(PET)_{18}]^-\cdot TOA^+$, the Polarizable Embedding Quantum Mechanics (PE-QM) framework implemented in DALTON[20] was employed. This approach enables the implicit inclusion of the PET ligands and the TOA^+ counterion as a polarizable embedding potential representing their electrostatic and polarization influence on the quantum mechanical part[21,22]. Initial AuNC--TOA^+ coordinates taken from the crystal structure were re-optimized using PBE and Grimme's D3 dispersion [23] to account for dispersion-driven noncovalent contacts between TOA^+ and the ligand shell. The resulting contact ion-pair geometry was then used for quadratic-response 2PA calculations as a proxy for tight ion pairing in toluene.

Fitting of the open aperture Z-scan curves in the case of competition between various processes contributing to nonlinear absorption

It is assumed that the overall extinction, including absorption and other losses like those due to scattering can be described by the generalized Lambert-Beer law:

$$\frac{dI}{dx} = -\alpha(I)I \qquad (1)$$

where I is the light intensity and $\alpha(I)$ is the intensity dependent extinction coefficient. In the current case we used the following expression for this coefficient:

$$\alpha(I) = \alpha_{unsat} + \frac{\alpha_0}{1+\frac{I}{I_{sat}}} + \alpha_2 I \qquad (2)$$

The terms on the right hand side of the equation denote the unsaturable part of the extinction coefficient, the saturable part where a simple rate equation derived expression is used, I_{sat} denoting the saturation intensity and the last term stands for two-photon absorption, α_2 being the two-photon absorption coefficient. It is apparent that the extinction coefficient measured for the system by an absorption spectrophotometer (i.e. at $I \cong 0$) should amount to $\alpha_{unsat} + \alpha_0$. It may be noted that the assumption that the low intensity extinction coefficient has two components, one of them saturable, the other one not saturable, may be necessary when one deals with a situation of overlap of two absorption bands, leading to two different excited states with different lifetimes and thus different saturation properties (very short lifetime of a state does not allow for the saturation).

The computation of the theoretical open aperture Z-scans proceeds as follows:

1) at each position z of the sample in the Z-scan setup the incident light intensity, at $x = 0$ is calculated as

$$I(z, r, x) = \frac{I_{max}}{1+\left(\frac{w_0}{w_z}\right)^2} e^{-2\left(\frac{r}{w_z}\right)^2} \qquad (3)$$

where I_{max} is the maximum light intensity (determined in a closed-aperture Z-scan experiment performed on a silica plate sample), r is the radial coordinate, w_0 is the focused beam spot radius at the focus ($z = 0$), also determined from the scan performed on silica, $w_z$is the beam radius at z, given by

$$w_z = w_0\sqrt{1 + \left(\frac{z}{z_R}\right)^2} \qquad (4)$$

where $z_R$is the Rayleigh length, $z_R = \frac{\pi w_0^2}{\lambda}$.

2) Equation (1) is numerically integrated over the thickness of the sample i.e. from $x = 0$ to $x = L$ for all positions of the sample and for concentring rings of the radius r from 0 to the arbitrary limit of $\frac{r}{w_z} = 4$
3) The transmittance is calculated at each z as

$$T(z) = \frac{\int_0^{4w_z} rI(z,r,L)dr}{\int_0^{4w_z} rI(z,r,0)dr} \qquad (5)$$

The computed curves were compared to the experimental ones, the parameters I_{max} and w_0 being fixed. The limited range of intensities (due to the damage to the sample at high intensities) and signal-to-noise considerations did not permit unequivocal determination of the parameter α_{unsat} , so the fitting was performed by allowing I_{sat} and α_2 to vary while $\alpha_0$was taken to be that obtained from spectrophotometer measurements and essentially equal to the value determined directly in the Z-scan setup by comparing the transmittance of the sample and that of pure solvent at positions far from $z = 0$.

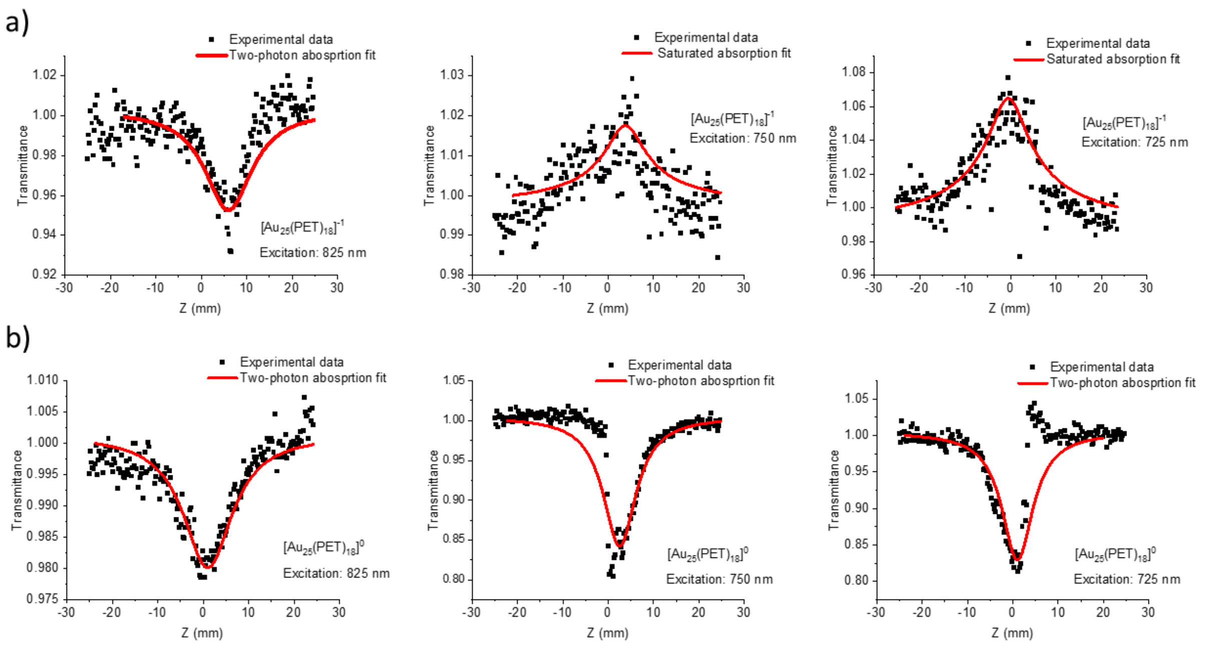

Figure S1. Representative nonlinear optical response of $Au_{25}(PET)_{18}$ as probed by the Z-scan technique at 725, 750 and 725 nm. a) Open-aperture (OA) Z-scan transmittance trace of $[Au_{25}(PET)_{18}]^{-1}$ and b) $[Au_{25}(PET)_{18}]^{0}$. Experimental data is presented with black dots, while red line corresponds to the theoretical fit – assuming two-photon absorption or saturated absorption (as indicated in graph legends). Scans were performed for the same sample concentration and the same excitation power 90 and 100 GW/cm^2.

Table S1. Summary of experimental two-photon absorption (σ_2) of Au_{25} nanoclusters. Values distinguished by absorption and emission-based techniques of measurements and supplemented by simulated σ_2.

Sample	σ_2 [GM]	λ[nm]	Technique	source
$Au_{25}(SR)_{18}^{-1}$ (hexane)	2 700 GM	1290 nm	TPEF	G. Ramakrishna et al. [24]
	427 000 GM	800 nm	two-photon excited time-resolved fluorescence upconversion	
$Au_{25}(SG)_{18}^{-1}$ (water)	4.99 GM	755 nm	TPEF	Russier-Antoine et al. [25]
$Au_{25}(SG)_{18}^{-1}$ (water)	189 740 GM	800nm	z-scan technique	L. Polavarapu et al.[26]
$Au_{25}(Capt)_{18}^{-1}$ (water)	23 800 GM/ 830 GM / 1510 GM	550 nm / 800 nm / 900 nm	z-scan technique	J. Olesiak-Banska et al. [7]
$Au_{25}(SH)_{18}^{-1}$	620 000 GM	1.58 eV (785nm)	TDDFT : B3LYP and a SDD-DZ basis set	P. N. Day et al. [27]
	753 GM	1.4 eV (885nm)	TDDFT: Becke–Perdew (BP86) XC and frozen-core triple-ζ polarized Slater-type (TZP) basis set)	Z. Hu et al. [28]
$Au_{25}(PET)_{18}^{-1}$ (toluene)	374 GM / 174 GM	900 nm / 1075 nm	z-scan technique	This work
$Au_{25}(PET)_{18}^{-0}$ (toluene)	7123 GM / 1819 GM / 1593 GM	825 nm / 900 nm / 1075 nm	z-scan technique	

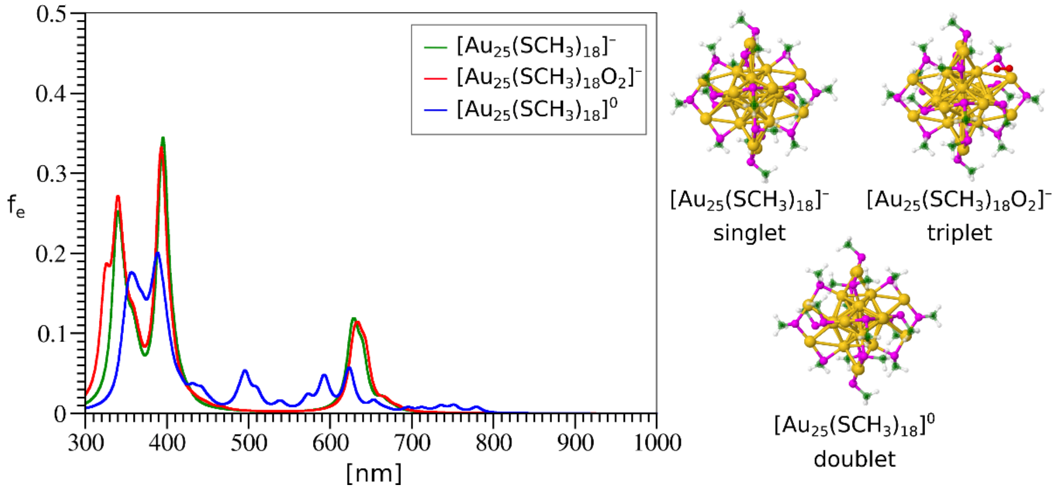

Figure S2. The TDDFT calculated 1PA spectra at CAM-B3LYP/def2-SVP level of theory of the $[Au_{25}(SCH_3)_{18}]^-$ (anion, singlet, green line), $[Au_{25}(SCH_3)_{18}O_2]^-$ (anion with one O_2, triplet, red line), and $[Au_{25}(SCH_3)_{18}]^0$ (neutral, doublet-unrestricted open shell, blue line). The corresponding structures are shown on the right with color coding: gold atoms in yellow, sulfur in magenta, carbon in green, oxygen in red, and hydrogen in white.

References

1 Parker, J. F., Weaver, J. E. F., McCallum, F., Fields-Zinna, C. A. & Murray, R. W. Synthesis of Monodisperse [Oct4N+][Au25(SR)18−] Nanoparticles, with Some Mechanistic Observations. *Langmuir* **26**, 13650-13654, doi:10.1021/la1020466 (2010).

2 Tofanelli, M. A. *et al.* Jahn–Teller effects in Au25(SR)18. *Chemical Science* **7**, 1882-1890, doi:10.1039/C5SC02134K (2016).

3 Zhu, M., Eckenhoff, W. T., Pintauer, T. & Jin, R. Conversion of Anionic [Au25(SCH2CH2Ph)18]− Cluster to Charge Neutral Cluster via Air Oxidation. *The Journal of Physical Chemistry C* **112**, 14221-14224, doi:10.1021/jp805786p (2008).

4 Sheik-Bahae, M., Said, A. A., Wei, T. H., Hagan, D. J. & Stryland, E. W. V. Sensitive measurement of optical nonlinearities using a single beam. *IEEE Journal of Quantum Electronics* **26**, 760-769, doi:10.1109/3.53394 (1990).

5 Samoc, M., Samoc, A., Luther-Davies, B., Humphrey, M. G. & Wong, M.-S. Third-order optical nonlinearities of oligomers, dendrimers and polymers derived from solution Z-scan studies. *Optical Materials* **21**, 485-488, doi:https://doi.org/10.1016/S0925-3467(02)00187-8 (2003).

6 Olesiak-Banska, J., Gordel, M., Kolkowski, R., Matczyszyn, K. & Samoc, M. Third-Order Nonlinear Optical Properties of Colloidal Gold Nanorods. *The Journal of Physical Chemistry C* **116**, 13731-13737, doi:10.1021/jp301821p (2012).

7 Olesiak-Banska, J., Waszkielewicz, M., Matczyszyn, K. & Samoc, M. A closer look at two-photon absorption, absorption saturation and nonlinear refraction in gold nanoclusters. *RSC Advances* **6**, 98748-98752, doi:10.1039/C6RA20610G (2016).

8 Perdew, J. P., Ernzerhof, M. & Burke, K. Rationale for mixing exact exchange with density functional approximations. *The Journal of Chemical Physics* **105**, 9982-9985, doi:10.1063/1.472933 (1996).

9 Perdew, J. P. *et al.* Atoms, molecules, solids, and surfaces: Applications of the generalized gradient approximation for exchange and correlation. *Physical Review B* **46**, 6671-6687, doi:10.1103/PhysRevB.46.6671 (1992).

10 Frisch, M. J. et al. Gaussian 16 Rev. C.01.) (2016)

11 Heaven, M. W., Dass, A., White, P. S., Holt, K. M. & Murray, R. W. Crystal Structure of the Gold Nanoparticle [N(C8H17)4][Au25(SCH2CH2Ph)18]. *Journal of the American Chemical Society* **130**, 3754-3755, doi:10.1021/ja800561b (2008).

12 Bhat, S. *et al.* Detection of [Au25(PET)18(O2)n]− (n = 1, 2, 3) Species by Mass Spectrometry. *The Journal of Physical Chemistry C* **122**, 19455-19462, doi:10.1021/acs.jpcc.8b03220 (2018).

13 Andrae, D., Häußermann, U., Dolg, M., Stoll, H. & Preuß, H. Energy-adjustedab initio pseudopotentials for the second and third row transition elements. *Theoretica chimica acta* **77**, 123-141, doi:10.1007/BF01114537 (1990).

14 Weigend, F. & Ahlrichs, R. Balanced basis sets of split valence, triple zeta valence and quadruple zeta valence quality for H to Rn: Design and assessment of accuracy. *Physical Chemistry Chemical Physics* **7**, 3297-3305, doi:10.1039/B508541A (2005).

15 Binkley, J. S., Pople, J. A. & Hehre, W. J. Self-consistent molecular orbital methods. 21. Small split-valence basis sets for first-row elements. *Journal of the American Chemical Society* **102**, 939-947, doi:10.1021/ja00523a008 (1980).

16 Dobbs, K. D. & Hehre, W. J. Molecular orbital theory of the properties of inorganic and organometallic compounds. 6. Extended basis sets for second-row transition metals. *Journal of Computational Chemistry* **8**, 880-893, doi:https://doi.org/10.1002/jcc.540080615 (1987).

17 Yanai, T., Tew, D. P. & Handy, N. C. A new hybrid exchange–correlation functional using the Coulomb-attenuating method (CAM-B3LYP). *Chemical Physics Letters* **393**, 51-57, doi:https://doi.org/10.1016/j.cplett.2004.06.011 (2004).

18 Kawasaki, H. *et al.* Generation of Singlet Oxygen by Photoexcited Au25(SR)18 Clusters. *Chemistry of Materials* **26**, 2777-2788, doi:10.1021/cm500260z (2014).

19 Norman, P. A perspective on nonresonant and resonant electronic response theory for time-dependent molecular properties. *Physical Chemistry Chemical Physics* **13**, 20519-20535, doi:10.1039/C1CP21951K (2011).

20 Aidas, K. *et al.* The Dalton quantum chemistry program system. *WIREs Computational Molecular Science* **4**, 269-284, doi:https://doi.org/10.1002/wcms.1172 (2014).

21 Olsen, J. M. H. & Kongsted, J. in *Advances in Quantum Chemistry* Vol. 61 (eds John R. Sabin & Erkki Brändas) 107-143 (Academic Press, 2011).

22 Olsen, J. M., Aidas, K. & Kongsted, J. Excited States in Solution through Polarizable Embedding. *Journal of Chemical Theory and Computation* **6**, 3721-3734, doi:10.1021/ct1003803 (2010).

23 Grimme, S., Antony, J., Ehrlich, S. & Krieg, H. A consistent and accurate ab initio parametrization of density functional dispersion correction (DFT-D) for the 94 elements H-Pu. *The Journal of Chemical Physics* **132**, doi:10.1063/1.3382344 (2010).

24 Ramakrishna, G., Varnavski, O., Kim, J., Lee, D. & Goodson, T. Quantum-Sized Gold Clusters as Efficient Two-Photon Absorbers. *Journal of the American Chemical Society* **130**, 5032-5033, doi:10.1021/ja800341v (2008).

25 Russier-Antoine, I. *et al.* Non-linear optical properties of gold quantum clusters. The smaller the better. *Nanoscale* **6**, 13572-13578, doi:10.1039/C4NR03782K (2014).

26 Polavarapu, L., Manna, M. & Xu, Q.-H. Biocompatible glutathione capped gold clusters as one- and two-photon excitation fluorescence contrast agents for live cells imaging. *Nanoscale* **3**, 429-434, doi:10.1039/C0NR00458H (2011).

27 Day, P. N., Nguyen, K. A. & Pachter, R. Calculation of One- and Two-Photon Absorption Spectra of Thiolated Gold Nanoclusters using Time-Dependent Density Functional Theory. *Journal of Chemical Theory and Computation* **6**, 2809-2821, doi:10.1021/ct100139t (2010).

28 Hu, Z. & Jensen, L. Importance of double-resonance effects in two-photon absorption properties of Au25(SR)18−. *Chemical Science* **8**, 4595-4601, doi:10.1039/C7SC00968B (2017).
